# The Importance of a Single Symptom

**Published:** 1883-05

**Authors:** Ad. Lippe

**Affiliations:** Philadelphia


### THE IMPORTANCE OF A SINGLE SYMPTOM.

AD. LIPPE, M. D., PHILADELPHIA.

The importance of a single symptom becomes apparent when we detect in a patient a single characteristic symptom corresponding with a single characteristic symptom observed in the proving of a drug. To illustrate this position I will, first, quote a case from my case-book in which an objective symptom indicated the truly specific remedy.

This case was one of very malignant "ship fever." The patient had been sick nine days when I found him in the morning, lying on his back, perfectly unconscious; eyes wide open, glaring, and fixed on the ceiling, pupils dilated; cheeks red and hot; mouth wide open, lower jaw hanging down; tongue and lips dry, black, and cracked; picking of bed coverings; pulse 200. The pathological condition was most certainly approaching paralysis of the brain.

The unconsciousness in this case reminded me at first of Bell.,

Hyos., Mur. ac., Opium, Rhus, or Stram. The eyes indicated Bell., Hyos., Opium, or Stram. The tongue and lips of Ars., Opium, or Rhus. The picking of the bed-clothes of Arn., Ars., Hyos., Opium, or Stram. The hanging of the lower jaw of Ars., Lyc., or Opium. Not being able to select a remedy, I further examined the patient and found that he had passed urine involuntarily all night, but this symptom again left me to choose between Arn., Ars., Bell., Hyos., or Rhus; but upon still further examination I found on the sheet of the patient a large deposit of red sand, resembling brick dust deposited from the urine involuntarily discharged. Here was the objective symptom characteristic of the case and of the remedy. I now concluded to give Lyc., therefore I dissolved six pellets of the 200th potency in half a tumblerful of water, and had a spoonful, every two hours, put into the open mouth of the unconscious patient. When I saw him again, at 2 P. M., I found him with his eyes and mouth firmly closed in a natural sleep and in a very heavy perspiration. He finally recovered fully, and enjoyed perfectly good health for many years.

In this case the single characteristic symptom was a guide in the selection of the curative remedy, but *not* in the diagnosis of the disease. If this single symptom had been a guide in the diagnosis of the disease, it would *not* have been a guide in the selection of the curative remedy.

To illustrate further the position assumed that one single symptom is very important, let us refer to the frequently recurring symptom, "sinking at the epigastrium." This symptom standing alone and by itself is of no importance whatever, neither characterizing a remedy or any abnormal condition of the system. Whether caused by a disturbed condition of pneumo-gastric nerve, or of the uterus, or by nervous depression, the symptom by itself, or the supposed cause, will never assist us in finding the curative remedy.

As far as our observations have been able to enlighten us, this symptom has been successfully removed by the following medicines: Alumen, Ambra, Baryta, Digitalis, Ignatia, Kali carb., Lobelia, Lycopodium, Petroleum, Oleander, Sepia, and Veratrum.

The important single symptom in this connection under Alumen is that the sinking sensation is aggravated after eating, while under Baryta it is relieved by eating. This symptom is on record in Hahnemann's *Chronic Diseases:* "Sensation of weakness in the stomach, which disappears after eating." The important single symptom under Ambra we find thus in Hahnemann's *Materia Medica Pura:* "*She* must lie down on account of giddiness and a sensation of weakness in the stomach."

Under Alumen and Baryta we find one conditional symptom, the aggravation and amelioration after eating. Under Baryta we find one concomitant and one conditional symptom, the combination of the sinking feeling with the condition of being obliged to lie down.

Digitalis has the characteristic symptom so often confirmed in practice and given by Hahnemann in his *Materia Medica Pura:* "A weakness of the stomach, as if the stomach were sinking away and as if life would vanish." Later it was observed that this sensation of weakness generally occurred "after eating."

Under Ignatia, we find in Hahnemann's *Materia Medica Pura:* "A peculiar sensation of weakness in the upper abdomen and in the pit of the stomach;" and "drawing and pinching in the lower abdomen, descending into the rectum like pressing, with qualmishness and sinking in the pit of the stomach and paleness of the face (after forty-eight hours, two days before menstruation)." And again: "Debility, as from weakness (sinking), around the pit of the stomach; *he* feels qualmish and must lie down."

Under Kali carb., we find in Hahnemann's *Chronic Diseases:* "Pressure in the stomach with rumbling, sensation of emptiness and eructations."

Under Laurocerasus, we find pain in the stomach, like fainting; feeling of weakness in the stomach.

Under Petroleum we find in Hahnemann's *Chronic Diseases:* "Sensation of emptiness in stomach, as from fasting."

Under Lobelia, we find: "Feeling of weakness of the stomach or in the pit of the stomach, extending through the whole chest."

Under Oleander, we find in Hahnemann's *Materia Medica Pura:* "Sensation of great emptiness in the pit of the stomach, with a fullness in the abdomen," and it has been observed that this sensation of emptiness in pit of stomach has been relieved by drinking brandy, often accompanied by nausea.

Under Sepia, we find in Hahnemann's *Chronic Diseases:* "Emptiness in the stomach (sensation of) with nausea as soon as she thinks of any food that might be offered to her."

Under Veratrum, we find in Hahnemann's *Materia Medica Pura:* "Sensation of weakness of the stomach, with an internal sensation of coldness in the region of the stomach and a light pressure."

The importance of a single symptom in connection with this much perplexing sensation of "sinking at the epigastrium," weakness at the pit of the stomach, is very obvious. We find that Alumen and Digitalis have an aggravation of this sensation after eating; that Baryta has an amelioration after eating; that under Oleander brandy relieves; that under Kali carb. is accompanied by eructations; that under Ignatia this sensation has appeared two days before menstruation, accompanied by pale face and qualmishness, which caused the patient to lie down; that under Sepia the sensation was increased by thinking of food. The sensation is strongest in the pit of the stomach under Digitalis, Ignatia, and Lobelia; under Digitalis the sensation is so intense that he feels as if life would vanish.

Many cases will be met in practice in which these symptoms are present with the sensation of sinking at the epigastrium; yet, at times, other remedies will have to be looked for to find symptoms corresponding with the peculiar characteristic symptoms of the patient.

A single symptom is all-important if it is the characteristic of the medicine, corresponding with the characteristic symptom of the case to be treated. Inasmuch as we no longer treat diseases, or supposed diseased conditions giving rise to them, but as we treat *patients*, it is no longer our duty to find the single symptom as a guide in diagnosis. It *is* our task, however, to find the single characteristic symptom both of the patient and of the remedy.

If we first get a clear idea of what constitutes the characteristics of medicines, we involuntarily adapt ourselves to the easy finding of the characteristic symptoms of the patient. The characteristic symptoms of a medicine go through all its pathogensis like a red streak. We find, for instance, that all the symptoms Aconite is capable of producing on the human system, and therefore is able to cure, are accompanied by "anxiety," and differ in the restlessness which is caused by "anxiety" under Aconite from the restlessness which is caused by "anguish" under Arsenic. Aconite has no characteristic pains. The burning and stinging in internal organs, tearing in external parts, and tingling in (fingers, œsophagus, and back) external parts, Aconite has in common with many other drugs; if, for instance, a patient complains of tearing in external parts, as in acute rheumatism, yet lies perfectly quiet, afraid to move, and if compelled to move suffers much pain, no experienced physician could think of administering Aconite, simply because the accompanying fever indicates inflammatory disease, but he would give Bryonia, if the other symptoms also indicated it. On the contrary, if the patient is very anxious and restless, not afraid to move, but tossing about, which he declares he cannot help, although it increases his pains, no one would give Bryonia, but Aconite, if otherwise indicated.

The "anxiety" of Aconite may be termed a general characteristic, like the "anguish" of Arsenic or the constant aggravation of all the symptoms after sleep under Lachesis, or the amelioration in open cold air under Pulsatilla; the amelioration the cold air alone being equally characteristic of Iodine, or the aggravation at 3 A. M. under Kali carb.

Besides these general characteristics which go through the whole remedy, we observe special characteristics, as under Kali bichr. that all the mucous discharges are stringy, or under Phosphorus that the cough is aggravated in the cold air.

The single symptom, which becomes all-important in a case, may comprise the kind of pains experienced, as under Apis "the burning, stinging pains;" or it may comprise the locality, as wrist and ankle under Ruta; or the direction the pain or disease follows, as from right to left, below upward, from the inside outward, or *vice versa;* or the condition (of amelioration or aggravation), as in the amelioration from heat of Arsenic, the amelioration from cold of Iodine; or from concomitant symptoms, as the great, unquenchable thirst, the great desire to drink large quantities under Natr. mur., or again, the thirstlessness of Pulsatilla.

The single symptom becomes all-important in some well-known diseases, as, for instance, in whooping cough. * * * * Yet the true physician has first to choose the proper remedy, and then to administer it properly if he hopes to be successful in this, as well as all other diseases. The character and peculiarities of the cough alone do not indicate a remedy. It is indispensably necessary to inquire further, and first ascertain at what time of the day the cough is aggravated. What else aggravates the cough? What are the concomitant symptoms? What is the character of the expectoration?

And in this manner it will become apparent that as to time the Drosera aggravation is after midnight; that the cough returning every day at the same hour may indicate either Lycopodium or Sabadilla.

Under the conditional aggravations it will become apparent that if pressure on the larynx aggravates the cough, Cina will be indicated; or that if walking fast brings on or aggravates the attack, Sepia will cure; or that if hasty eating or drinking causes an attack, Silicea will cure; or, with regard to the expectoration, that if the great quantity of mucus which threatens to suffocate the patient is difficult to expectorate, and if raised at all is tough and stringy and hard to detach, etc., Coccus cacti is the remedy.

All these single symptoms become important and will enable the practitioner to select the curative remedy; the name of the disease never will, as no medicine has ever produced or can produce whooping cough, but only a cough similar to whooping cough. The cough produced by Mephitis, for instance, has been very similar to whooping cough, but was not whooping cough and can only cure in those cases where the concomitant symptoms correspond with Mephitis.—*Am. Hom. Review*, 1863.

---

This paper was written twenty years ago, and after that long lapse of time its author is more than ever convinced of the great importance of the single symptom. The single peculiar symptom, expressive, as it were, of the characteristic individuality of the sick and not necessarily belonging to the form of disease of which he suffers, if also characteristic of a proved drug—becomes very frequently a guiding symptom, will very often lead us to compare the symptoms of the sick with the symptoms of the drug presenting that guiding symptom, a remedy which probably escaped our notice without it, and if the similarity between the symptoms of the sick and the provings of the drug become apparent, *then* and *then only* has this single guiding symptom been profitably utilized. Later on it was claimed that this single symptom, when present both on the sick and in the provings, would absolutely demand recognition and was erroneously termed a key-note, and this erroneous interpretation of the importance of the single symptom opened the way for great and fatal abuses. And now for an illustration: We find, for instance, in that excellent work on Diphtheria by Dr. Gregg, a case of diphtheria cured by Lachnanthes. The indications for the use of Lachnanthes were—the stiff neck the patient had. A cure followed. The deduction from this observation of a cure would be that a stiff neck in diphtheria is a key-note for Lachnanthes in that disease. This is, of course, poor logic, and later experience illustrates it. In a case of diphtheria we lately published this very painful stiff neck was a very prominent symptom, and the clinical experiment has shown that another case of diphtheria where this stiff neck was present had been promptly cured by Lachnanthes, that in the stiff neck following not unfrequently diphtheria and scarlet fever Lachnanthes has very often cured it. In the case alluded to, all and every symptom of the patient suffering from diphtheria, also the stiff neck, were covered by Kali bichr., and the stiff neck was cured with the other very grave symptoms of diphtheria. Kali bichr. has probably never been given for wryneck before, and now if in a case of diphtheria this stiff neck occurs, we will have to take into consideration the similarity of the other symptoms of the patient, having this guiding symptom to make us compare Kali bichr. and Lachnanthes. Again, we find under Lachesis, as a very characteristic symptom, throat and cough symptoms worse after awaking. The clinical experiment demonstrates that Kali bichr. and Aralia have the same symptom, and we will now, knowing that this great aggravation after sleeping is not a key-note for Lachesis, not be easily disappointed when we carefully compare also other remedies causing and curing this single important symptom. The lesson we are taught is, that a single important symptom *alone* should not be termed a key-note, but a guiding symptom.

